# Clinical research updates

**DOI:** 10.1111/camh.70111

**Published:** 2026-07-15

**Authors:** Marinos Kyriakopoulos, Vasileia Liakou, Lydia‐Dimitra Stavropoulou, Odysseus Malafouris

**Affiliations:** ^1^ National and Kapodistrian University of Athens Athens Greece; ^2^ Department of Child and Adolescent Psychiatry, Institute of Psychiatry Psychology and Neuroscience King's College London London UK

## Associations between infant social withdrawal and disorganized attachment

Vasileia Liakou

National and Kapodistrian University of Athens

Disorganized attachment constitutes a significant risk factor for the development of psychopathology, low social competence, and difficulties in school adjustment. Given that its assessment in infants younger than 11 months is not reliable, the identification of predictive factors earlier in development is paramount.

Stuart et al. (2026) investigated two such factors, namely infant social withdrawal and maternal postpartum depression, in a sample of 203 Danish mother–infant dyads. The aim of the study was to examine whether maternal postpartum depressive symptoms and infant social withdrawal, as assessed when the infants were 2–11 months old, were associated with disorganized attachment at 11–20 months of age. As part of a broader study on infant mental health in Denmark, health visitors assessed mothers and infants and depending on their scores, referred them to the study, after the evaluation by a psychologist. When the infants were 11–20 months old, attachment in the mother–child relationship was investigated. The Edinburgh Postnatal Depression Scale (EPDS) was used to assess postpartum depression, and the Alarm Distress Baby Scale (ADBB) was used to assess infant social withdrawal. The Strange Situation Procedure (SSP) experimental tool was used to examine attachment quality across eight short episodes, with scores ranging from 1 to 9. Scores above 5 were considered indicative of disorganized attachment. Statistical analyses of the variables were conducted using *R* software with *p* = .05, while the influence of potential confounding factors was also examined.

The study found that boys were more likely to exhibit disorganized attachment. Maternal EPDS scores were not significantly associated with disorganized attachment; however, compared with infants exhibiting secure and organized attachment, infants displaying social withdrawal were 1.21 times more likely to exhibit disorganized attachment. The lack of association found with maternal depressive symptoms may be due to limited variability of symptoms in the sample and the support available to participants. The relationship between social withdrawal and attachment may be explained through the pathways of ‘threat conflict’ and ‘ambiguity of the secure base’, according to Bowlby. Such experiences may lead infants to develop a generalized pattern of dysregulation, where withdrawal functions as a strategy for regulating intense emotions.

Limitations of the current study include the examination of an at‐risk population, the relatively small sample size, and the fact that some mothers received a parenting intervention which could have influenced attachment. Overall, this study is the first to provide empirical support that persistent social withdrawal in infants is associated with disorganized attachment, opening the way for more systematic and longitudinal investigations of the relationship between these two variables.

Stuart, A. C., Røhder, K., Reijman, S., Egmose, I., Smith‐Nielsen, J., Madsen, E. B., … & Vaever, M. S. (2026). Associations between infant social withdrawal and disorganized attachment. *European Child & Adolescent Psychiatry*. https://doi.org/10.1007/s00787‐026‐03057‐9

## Self‐blame as a mediator between problematic social media use and suicidal ideation in adolescents

Lydia‐Dimitra Stavropoulou

National and Kapodistrian University of Athens

The increasing prevalence of problematic social media use (PSMU) has raised concerns about its adverse impact on mental health, particularly suicidality. Adolescents constitute a vulnerable population due to their greater propensity for sensation‐seeking, impulsive, and risky behaviors, as well as their heightened sensitivity to social evaluation.

Quintana‐Orts et al. (2026) conducted a study that employed a prospective design over a 9‐month period to examine the mediating role of four specific maladaptive cognitive emotion regulation strategies (CERS), namely rumination, self‐blame, other‐blame, and catastrophizing, in the relationship between PSMU and suicidal ideation in adolescents. Their sample consisted of 517 students enrolled in grades 7 through 10 evaluated at 2 time points. The Spanish Version of Bergen Social Media Addiction Scale (BSMAS) was used to evaluate PSMU symptoms, the Frequency of Suicidal Ideation Inventory (FSII) measured the frequency of suicidal thoughts, and the Cognitive Emotion Regulation Questionnaire for adolescents (CERQ‐SA) assessed cognitive coping mechanisms. In addition, the adapted short form of Goldberg's bipolar adjectives was used to evaluate personality attributes.

This study found a positive prospective association between PSMU and suicidal ideation 9 months later, a finding that aligns with existing evidence on the detrimental effects of excessive use of social media on mental health and, more specifically, on the risk of suicide. Furthermore, PSMU was positively associated with maladaptive CERS, supporting previous findings that link emotion regulation difficulties to externalizing problems and problematic behaviors. Maladaptive CERS were prospectively correlated with suicidal ideation, with self‐blame showing the strongest correlation, followed by catastrophizing and rumination. Self‐blame emerged as the only significant mediator between PSMU and suicidal ideation. An unexpected result, however, was the lack of a prospective association between other‐blame and suicidal ideation. This finding is explained by the fact that internally oriented cognitive processes, including self‐blame, self‐criticism, and rumination, have been associated with internalizing psychopathology in adolescence. In contrast, externally oriented attributional tendencies, such as hostile attribution or externalization of blame, have been more strongly associated with externalizing outcomes, including aggression and disruptive behavior.

Several limitations have been identified. Other than suicidal ideation, suicide attempts or behaviors and nonsuicidal self‐injury were not specifically examined. In addition, the study did not account for other relevant affective disorders that may function as confounding variables or mediators between PSMU and self‐harm behavior, relied solely on self‐reported measures, and reverse causality cannot be ruled out.

Quintana‐Orts, C., Yudes, C., Sánchez‐Moreno, V., & Rey, L. (2026). When social media hurts: a nine‐month prospective study on self‐blame as a mediator between problematic social media use and suicidal ideation in adolescents. *European Child and Adolescent Psychiatry*. https://doi.org/10.1007/s00787‐026‐03015‐5

## The psychosocial impacts of autism‐related genetic testing

Odysseus Malafouris

National and Kapodistrian University of Athens

Growing clinical and research interest in identifying the genetic underpinnings of autism spectrum disorder (ASD) has encouraged a parallel increase in clinical guidance for parents to have their children undergo genetic testing. Despite this, there is still a notable gap in the literature when it comes to investigating the psychosocial repercussions for both the parents and autistic individuals following the disclosure of genetic testing outcomes.

Willis et al. (2026) conducted a systematic review to examine the emotional and behavioral consequences of ASD‐related genetic testing while addressing the relevant ethical and equity challenges in informed decision making. The authors searched MEDLINE, PsycINFO, CINAHL, Web of Science Core Collection, Sociological Index, and Scopus for articles on human‐subjects research in the English language, published from 1990, after the launch of the Human Genome Project, to 2024. Methodological thresholds required a minimum sample size of 20 participants for quantitative studies and 5 for qualitative studies. The review excluded papers focused solely on pretest attitudes or broad syndromic conditions where autism is merely a secondary feature (such as Fragile X syndrome), isolating only those that captured the psychological impacts of actual or hypothetical autism‐specific outcomes. A total of 3606 articles were screened, 22 of which (18 of actual and four of hypothetical genetic test results) met the eligibility criteria for inclusion. Of these, two articles assessed impacts on autistic adults. The quality of these studies was appraised using the QuADS tool to synthesize cognitive, emotional, and behavioral impacts across both actual and hypothetical testing.

Findings regarding the psychological consequences for parents revealed an impact profile of mixed valence and modest magnitude. Responses ranged from emotional relief and dampened guilt (particularly in the cases of identifiable de novo mutations) to disappointment, anxiety, and fear stemming from residual clinical uncertainty. Genetic testing outcomes were found to frequently influence family planning and prenatal decision‐making, including decisions regarding pregnancy continuation or termination. As such, the significance of this review is grounded in the advocacy for robust pre‐ and post‐test genetic counseling, especially as a substantial discrepancy was identified between parents' high expectations and the actual clinical utility of the test results. Furthermore, the authors highlight ethical issues related to concerns of autistic adults regarding autonomy, discrimination, and testing without explicit consent.

As is the case with most research of this nature, a primary limitation of the reviewed literature is the substantial heterogeneity in study designs, assessment methods, and outcome measures. Additionally, the existing body of research suffers from a noteworthy lack of demographic diversity, as sample populations were predominantly White mothers, leaving the direct, long‐term experiences of racially diverse families and autistic individuals themselves significantly underrepresented.

Wills, B. C., Matthews, M. M., Johnston, J., Bolo, I., Ottman, R., & Appelbaum, P. S. (2026). Systematic review: The psychosocial impacts of autism‐related genetic testing. *Journal of the American Academy of Child & Adolescent Psychiatry*, 65(4), 505–526. https://doi.org/10.1016/j.jaac.2025.06.024

## Funding information

No funders declared.

## Conflict of interest statement

M.K. is the *CAMH* Associate Editor for Clinical Research Updates. The editor thanks the contributors for this issue's Clinical Research Updates. The editor has declared that he has no competing or potential conflicts of interest in relation to this article, and all remaining authors declare no competing or potential conflicts of interest.

## Ethics statement

No ethical approval was required for these updates.

## Data Availability

Data sharing is not applicable to this article as no new data were created or analyzed for these updates.

